# Enzymatic fluorometric assays for quantifying all major phospholipid classes in cells and intracellular organelles

**DOI:** 10.1038/s41598-019-45185-0

**Published:** 2019-06-13

**Authors:** Tokuji Tsuji, Shin-ya Morita, Yoshito Ikeda, Tomohiro Terada

**Affiliations:** Department of Pharmacy, Shiga University of Medical Science Hospital, Otsu City, Shiga 520-2192 Japan

**Keywords:** Phospholipids, Phospholipids, Organelles

## Abstract

Cell membrane phospholipids regulate various biological functions. We previously reported enzymatic fluorometric methods for quantifying phosphatidic acid, phosphatidylcholine, phosphatidylethanolamine, phosphatidylserine, sphingomyelin, phosphatidylglycerol and cardiolipin. In the present report, a new enzymatic fluorometric assay was developed for quantifying phosphatidylinositol. These simple, sensitive and high-throughput methods enabled us to quantify all major phospholipid classes in cultured cells and intracellular organelles. By conducting comprehensive quantitative analyses of major phospholipid classes, we demonstrated that the contents of phospholipid classes in HEK293 cells changed with cell density and that overexpression of phosphatidylinositol synthase or CDP-diacylglycerol synthase significantly affected the phospholipid compositions of microsomal and mitochondrial membranes. These enzymatic fluorometric assays for measuring all major phospholipid classes may be applicable to tissues, fluids, lipoproteins, extracellular vesicles and intracellular organelles of many organisms and will further our understanding of cellular, physiological and pathological processes.

## Introduction

Phospholipids are essential structural components of cell membranes and plasma lipoproteins, and are involved in numerous biological responses, including membrane protein regulation, membrane trafficking, apoptosis, cellular signalling and lipoprotein metabolism^1–9^. Phospholipid molecules consist of a hydrophilic head group and a hydrophobic region containing two acyl chains, and are divided into two groups, glycerophospholipids and sphingophospholipids, based on the backbone structure. Glycerophospholipids are further classified by the structures of the head group into classes, including phosphatidylinositol (PI), phosphatidic acid (PA), phosphatidylcholine (PC), phosphatidylethanolamine (PE), phosphatidylserine (PS), phosphatidylglycerol (PG) and cardiolipin (CL). In mammalian cells and plasma lipoproteins, the most abundant sphingophospholipid is sphingomyelin (SM)^9^. In addition, there are over thousands of phospholipid molecular species differing in acyl chain composition^4^. In mammalian cells, phospholipids are produced mainly in the endoplasmic reticulum (ER) and mitochondria through pathways involving many enzymes and are then translocated to other organellar membranes^3,4,7,8^. Previous studies have reported that disturbances in phospholipid metabolism are closely associated with diverse disorders, including cancer, autoimmune disease, dyslipidaemia and atherosclerosis^1,5,9^.

The conventional assay for measuring phospholipid classes involves thin-layer chromatography (TLC) separation and subsequent phosphate quantification from the spots. Although the phospholipid compositions of various animal tissues have been evaluated for over 50 years using TLC methods^10^, it has been difficult to measure low levels of phospholipid classes in cultured cells or intracellular organelles. The evaporative light-scattering detector for high-performance liquid chromatography has been applied to quantify phospholipid classes, including PI^11^. Mass spectrometry is very useful for the characterization of phospholipid molecular species differing in acyl chain composition^12–14^. Electrospray ionization mass spectrometry has been successfully used for detecting, identifying and quantifying PI and phosphatidylinositol phosphates (PIPs) with different acyl chain compositions under conditions in which the ion response does not vary substantially over the investigated mass range^15^. However, in general, correction curves are required to quantify each phospholipid molecular species because of differences in the ionization efficiency among phospholipid species with different fatty acyl chains. Thus, the development of new methodologies for quantifying cellular and organellar phospholipid classes is highly desirable to clarify phospholipid functions and metabolism. We therefore recently developed enzyme-based fluorometric methods to quantify PA, PC, PE, PS, the sum of PG and CL (PG + CL), and SM^13,16–19^. However, there is no simple and sensitive assay for PI measurement.

In mammalian cells, PI is biosynthesized from *myo*-inositol and CDP-diacylglycerol (CDP-DAG) by phosphatidylinositol synthase (PIS) in the ER and highly mobile ER-derived membrane compartments^20–24^. CDP-diacylglycerol synthase 1 (CDS1) and CDS2, localized in the ER, catalyse the conversion of PA to CDP-DAG, a pivotal intermediate in the biosyntheses of phospholipid classes including PI^22,23,25–27^. It has been reported that CDS1 localizes to nonraft microdomains of the ER enriched for calnexin^28^. In addition, PI is phosphorylated to form PIPs, such as PI(4)P, PI(4,5)P_2_ and PI(3,4,5)P_3_, which are involved in many cellular functions such as growth and vesicular trafficking^6^. Nevertheless, little is known about the relationships between cellular functions and quantitative changes in the PI content in cellular membranes. Therefore, in the present study, we developed a fluorometric assay for quantifying PI using specific enzymes to complement the methods for measuring PA, PC, PE, PS, PG + CL and SM. These enzymatic assays allow simple, sensitive and high-throughput measurement of all major phospholipid classes and can be routinely used for determining the amounts of phospholipid classes in cells and intracellular organelles. Furthermore, using these novel methods, we assessed the relationship of cellular phospholipid compositions with cell density and investigated the effects of PIS, CDS1 and CDS2 overexpression on the phospholipid composition in cells and intracellular organelles.

## Results and Discussion

### Enzymatic fluorometric assay for PI measurement

**Figure 1:**
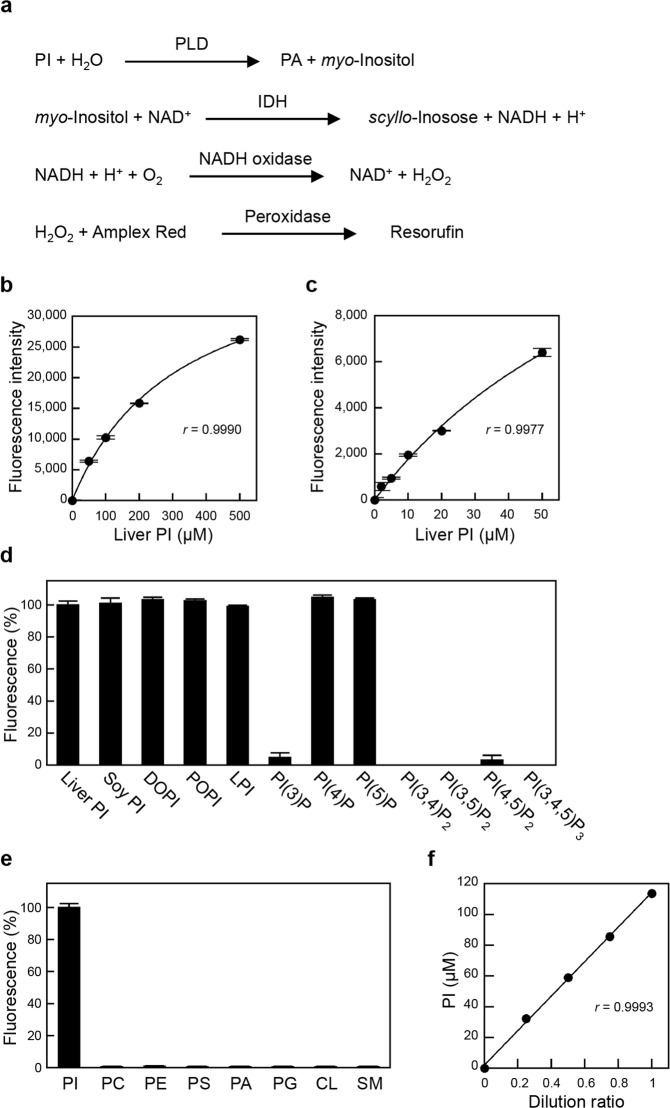
Enzymatic measurement of PI. (**a**) Strategy for PI measurement. PLD catalyses the hydrolysis of PI to PA and *myo*-inositol. IDH catalyses the oxidation of *myo*-inositol and the reduction of NAD^+^. NADH is oxidized by NADH oxidase, which produces hydrogen peroxide. In the presence of peroxidase, Amplex Red reacts with hydrogen peroxide to produce highly fluorescent resorufin. (**b**,**c**) Standard curves for PI measurement using liver PI standard solutions. The background fluorescence was 7019 ± 107, which was subtracted from each value. Each point represents the mean ± S.D. (n = 3). The lines were obtained by hyperbolic regression analysis. The correlation coefficients were *r* = 0.9990 at higher concentrations (**b**) and *r* = 0.9977 at lower concentrations (**c**). (**d**) Fluorescence changes in response to 100 μM liver PI, soy PI, DOPI, POPI, LPI, PI(3)P, PI(4)P, PI(5)P, PI(3,4)P_2_, PI(3,5)P_2_, PI(4,5)P_2_ and PI(3,4,5)P_3_. (**e**) Fluorescence changes in response to 100 μM liver PI, egg PC, liver PE, brain PS, egg PA, egg PG, heart CL and egg SM. The fluorescence increase in response to liver PI was taken as 100%. Each bar represents the mean ± S.D. (n = 3). (**f**) Linearity of PI measurement. The lipid extract from HEK293 cells was sequentially diluted with 1% Triton X-100. The correlation coefficient was *r* = 0.9993.

We developed a novel assay for measuring PI, involving a four-step enzymatic reaction (Fig. 1a). (1) Phospholipase D (PLD) hydrolyses PI to PA and *myo*-inositol. (2) *myo*-Inositol dehydrogenase (IDH) catalyses the oxidation of *myo*-inositol and the reduction of NAD^+^, which generate *scyllo*-inosose and NADH, respectively. (3) NADH is oxidized by NADH oxidase to produce NAD^+^ and H_2_O_2_. (4) H_2_O_2_ is reacted with Amplex Red in the presence of peroxidase to generate resorufin, which is highly fluorescent and measurable. Amplex Red has been used to measure the concentrations of H_2_O_2_ and NADH^29,30^. This method requires only 10 μl of sample in a 96-well plate format.

We performed a calibration reaction using liver PI standard solutions to validate this new enzymatic PI assay. At higher and lower concentrations, the calibration curve was less linear and fit a hyperbolic regression equation (*r* = 0.9990 and *r = *0.9977, respectively), and the detection limit was as low as 2 μM (20 pmol in the reaction mixture) (Fig. 1b,c). As shown in Fig. 1d, the fluorescence change in response to soy PI, 1,2-dioleoyl PI (DOPI) or 1-palmitoyl-2-oleoyl PI (POPI) was the same as that in response to liver PI containing mixed acyl chains when normalized to the moles. Therefore, neither the length of the acyl chain nor the number of double bonds influence the PI assay. There was no difference in the fluorescence changes between liver PI and lysophosphatidylinositol (LPI), indicating that *Streptomyces chromofuscus* PLD can release *myo*-inositol from PI and LPI. PI(4)P and PI(5)P also increased the fluorescence intensity to the same extent as liver PI (Fig. 1d). Thus, this measurement cannot distinguish among PI, LPI, PI(4)P, and PI(5)P. However, PI(3)P and PI(4,5)P_2_ exhibited only negligible fluorescence increases (<5% compared with that induced by liver PI), and PI(3,4)P_2_, PI(3,5)P_2_ and PI(3,4,5)P_3_ showed no increase in fluorescence (Fig. 1d). In addition, the other phospholipid classes, PC, PE, PS, PA, PG, CL and SM, were not detected by the PI assay (Fig. 1e).

### Quantification of PI in cultured cells

*myo*-Inositol, NADH and H_2_O_2_ are contained in cultured cells and confound the enzymatic measurement of PI. Hence, the removal of contaminating *myo*-inositol, NADH and H_2_O_2_ from samples was necessary for the enzymatic assay. The method of Folch is effective for the extraction of a broad range of lipid classes, including PI, and has been broadly applied for lipid extraction to quantify phospholipids by mass spectrometry or TLC^10,31,32^. In the method of Folch using chloroform, methanol and water, *myo*-inositol, NADH and H_2_O_2_ partition into the upper aqueous phase whereas phospholipids partition into the lower organic phase. We assessed how effectively *myo*-inositol, NADH and H_2_O_2_ can be removed from samples using the method of Folch. Enzymatic fluorometric methods have been developed for determining NADH and H_2_O_2_ concentrations^19,29,30^, and we modified the enzymatic assay for quantifying *myo*-inositol by using Amplex Red^33^. The calibration curves for enzymatic fluorometric measurement of *myo*-inositol were hyperbolic and quadratic at high and low concentrations, respectively (Supplementary Fig. S1a,b). At high and low concentrations of NADH, the curves fit linear and quadratic regression equations, respectively (Supplementary Fig. S2a,b). The calibration curves for H_2_O_2_ measurement were linear (Supplementary Fig. S3a,b). Although the samples contained 10–500 μM *myo*-inositol, NADH or H_2_O_2_ before lipid extraction, the samples contained no detectable *myo*-inositol, NADH or H_2_O_2_ (<1 μM) after lipid extraction (Supplementary Figs S1c, S2c and S3c). These results demonstrated that the high concentration of *myo*-inositol, NADH or H_2_O_2_ in samples can be completely removed by the Folch procedure. To avoid contamination by *myo*-inositol, NADH and H_2_O_2_, the extraction of lipids from cells is recommended for this PI assay. PIPs and LPI are well-known cell signalling mediators, but, in general, their amounts in cell membranes are much lower than those of PI^6,34,35^. Although PI(4)P constitutes only 2–5% of PI in typical mammalian cells, the Golgi contains very high levels of PI(4)P^35^. However, acidification is required to efficiently extract PIPs and LPI by the Folch procedure^15,36^. Wenk *et al*. reported that after neutral (nonacidified chloroform-methanol) extraction, the lipid extracts from brain tissues are substantially devoid of PIPs^15^. We also evaluated the effects of the nonacidified Folch extraction method on the PI(4)P amount in the sample. Using the enzymatic fluorometric assay for PI, we detected no PI(4)P in the sample after lipid extraction by the method of Folch (Supplementary Fig. S4), indicating the almost complete removal of PI(4)P. Therefore, after extraction by the standard Folch method, the concentrations of PI(4)P, PI(5)P and LPI may be negligible compared with that of PI in the cellular phospholipid extract.

To confirm the accuracy of this assay, we carried out a recovery test in which known quantities of liver PI were added to the lipid extract from HEK293 cells. At concentrations of 25–250 μM, the mean recovery of PI was 99.8% (Supplementary Table S1), indicating that no hydrophobic interfering compounds were extracted from the cells. Next, to assess the linearity of this PI measurement, the cellular lipid extract was serially diluted with 1% Triton X-100 aqueous solutions. Figure 1f shows a well-fitted regression line to 113.7 μM PI (*r* = 0.9993). At least 3.0 × 10^5^ cells were required to measure the cellular PI content using this enzymatic fluorometric assay. Collectively, these data demonstrate that this novel enzymatic PI assay is sufficiently accurate and sensitive to detect changes in endogenous cellular PI levels.

### Relationships between cell density and PI content in HEK293 cells

**Figure 2:**
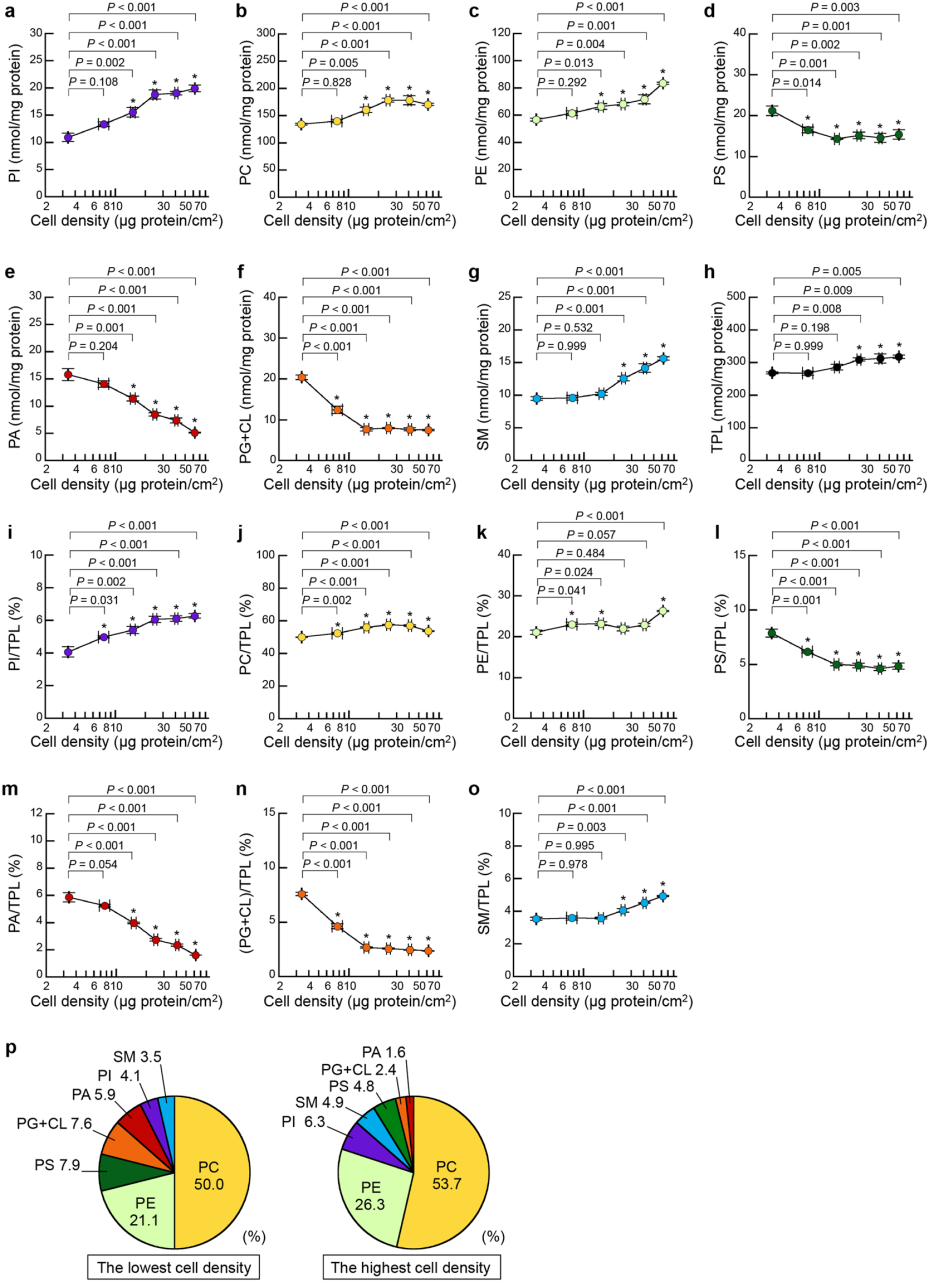
Effects of cell density on phospholipid composition in HEK293 cells. HEK293 cells were seeded in 10-cm dishes at varying cell densities and were cultured in MEM containing 10% FBS for 48 h at 37 °C. Then, the cells were incubated with MEM supplemented with 0.02% BSA for 18 h at 37 °C. After incubation, cellular phospholipids were extracted. The contents of PI (**a**), PC (**b**), PE (**c**), PS (**d**), PA (**e**), PG + CL (**f**) and SM (**g**) at varying cell densities were determined by the enzymatic measurements and protein assay. The total phospholipid (TPL) content (**h**) was calculated as the sum of PI, PC, PE, PS, PA, PG + CL and SM contents. The ratios of PI/TPL (**i**), PC/TPL (**j**), PE/TPL (**k**), PS/TPL (**l**), PA/TPL (**m**), (PG + CL)/TPL (**n**) and SM/TPL (**o**) at varying cell densities were calculated (mean ± S.E., n = 3, *P < 0.05, significantly different from in HEK 293 cells at the lowest cell density (3.38 ± 0.03 μg protein/cm^2^), one-way ANOVA followed by Dunnett’s test, *df* = 17, (**a**) *F* = 27.81, (**b**) *F* = 18.66, (**c**) *F* = 23.79, (**d**) *F* = 7.86, (**e**) *F* = 50.00, (**f**) *F* = 112.08, (**g**) *F* = 47.25, (**h**) *F* = 7.29, (**i**) *F* = 17.88, (**j**) *F* = 68.71, (**k**) *F* = 15.07, (**l**) *F* = 28.89, (**m**) *F* = 126.21, (**n**) *F* = 289.11, (**o**) *F* = 49.71). (**p**) The phospholipid compositions in HEK 293 cells at the lowest cell density and at the highest cell density (62.20 ± 0.50 μg protein/cm^2^) are shown as pie charts.

Many cell functions, as well as membrane morphology, are affected by cell density. Using enzymatic assays for the phospholipid classes, we previously found that the cellular contents of major phospholipid classes, including PC, PE, PS, PA, PG + CL and SM, changed depending on cell density^13,16–19^. PI is the precursor of PIPs, which are involved in various cellular processes, including growth, proliferation, elongation, and migration^6,37^. Thus, the cellular content of PI is expected to be highly regulated with cell growth. However, due to the difficulty in measuring the PI content in sparse cell cultures by previous methods using TLC, it remains unclear whether the change in cell density affects the cellular PI content. Using the novel enzymatic assay, we measured the PI content in HEK293 cells at varying cell densities. Supplementary Fig. S5 shows images of HEK293 cells at varying cell densities ranging from 2.91 to 79.75 μg protein/cm^2^. The cellular PI content increased with increasing cell density and became constant between the medium and highest cell densities (24.97 ± 1.14 and 62.20 ± 0.50 μg protein/cm^2^, respectively) (Fig. 2a). Consistent with our previous results^13,16–19^, the cellular contents of PC, PE and SM increased in a cell density-dependent manner, whereas the contents of PS, PA and PG + CL decreased with increasing cell density (Fig. 2b–g). The total phospholipid (TPL) contents at higher cell densities (ranging from 24.97 ± 1.14 to 62.20 ± 0.50 μg protein/cm^2^) were greater than those at lower cell densities (from 3.38 ± 0.03 to 7.71 ± 0.90 μg protein/cm^2^) (Fig. 2h). We also evaluated the content ratio of each phospholipid class to TPL at varying cell densities. The PI/TPL ratio increased with increasing cell density (Fig. 2i). Additionally, the increase in the cell density was accompanied by increases in the PC/TPL, PE/TPL and SM/TPL ratios but by reductions in the PS/TPL, PA/TPL, and (PG + CL)/TPL ratios (Fig. 2j–o). Moreover, the phospholipid compositions were compared at the lowest cell density and the highest cell density (3.38 ± 0.03 and 62.20 ± 0.50 μg protein/cm^2^, respectively). At both cell densities, the most abundant and the second most abundant cellular phospholipids were PC and PE, respectively (Fig. 2p). Notably, the amount of PI was higher than the amount of PS, PG + CL or PA at the highest cell density but not at the lowest cell density. These observations suggest that the amounts of all membrane phospholipid classes, including PI, are dynamically controlled by signals from cell maturation, proliferation, migration and/or cell-cell interactions.

### Effects of PIS, CDS1 or CDS2 overexpression on the phospholipid composition in HEK293 cells

**Figure 3:**
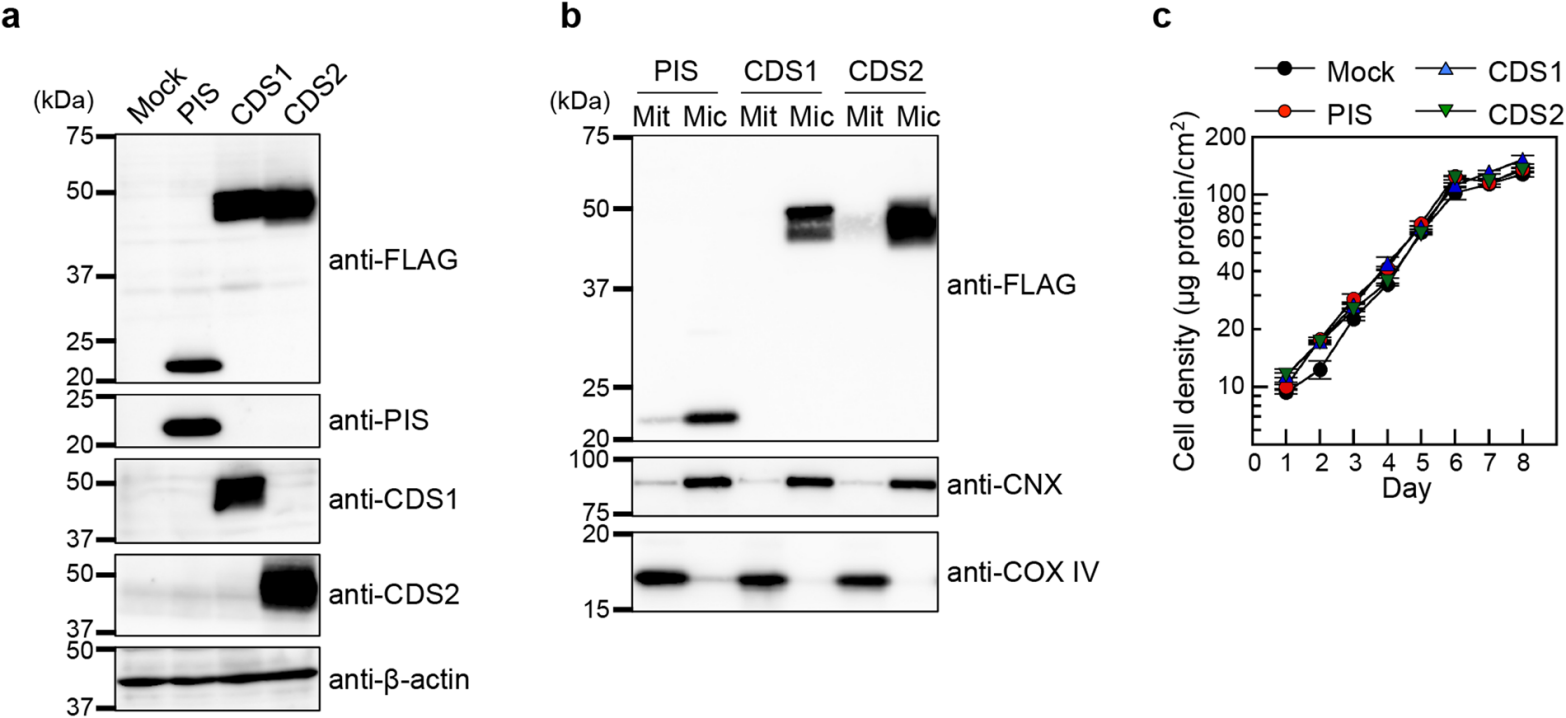
Establishment of HEK293 cells stably overexpressing PIS, CDS1 or CDS2. (**a**) Expression of FLAG-PIS, FLAG-CDS1 and FLAG-CDS2 in HEK293 cells. Whole cell lysates (20 μg of protein) from HEK293 mock (Mock), HEK/FLAG-PIS (PIS), HEK/FLAG-CDS1 (CDS1) and HEK/FLAG-CDS2 (CDS2) cells were separated by 10% SDS-PAGE, and then immunoblotted with anti-FLAG, anti-PIS, anti-CDS1, anti-CDS2 or anti-β-actin antibody. (**b**) Isolation of purified microsomal (Mic) and mitochondrial (Mit) fractions from HEK/FLAG-PIS, HEK/FLAG-CDS1 and HEK/FLAG-CDS2 cells. The purified microsomal and mitochondrial fractions (2.0 μg of protein) were separated by 7%, 10% or 15% SDS-PAGE and then immunoblotted with anti-FLAG, anti-CNX or anti-COX IV antibody. CNX and COX IV are markers of the ER and mitochondria, respectively. The full-length blots are presented in Supplementary Fig. S6. (**c**) Effects of PIS, CDS1 or CDS2 overexpression on cell proliferation. HEK293 mock (black circles), HEK/FLAG-PIS (red circles), HEK/FLAG-CDS1 (blue triangles) and HEK/FLAG-CDS2 (green inverted triangles) cells were cultured in six-well plates in MEM containing 10% FBS at 37 °C for 8 days. The doubling times between days 1 and 6 were not significantly different among these cell lines (HEK293 mock, 35.03 ± 1.23; HEK/FLAG-PIS, 33.21 ± 0.05; HEK/FLAG-CDS1, 36.49 ± 0.46; HEK/FLAG-CDS2, 35.16 ± 0.38 h, mean ± S.E., n = 3, one-way ANOVA, *df* = 11, *F* = 3.92, *P* = 0.054).

In the ER of mammalian cells, PIS, CDS1 and CDS2 are key enzymes in PI biosynthesis. CDP-DAG is produced from PA by the action of CDS1 and CDS2, which are 461- and 445-amino acid membrane proteins, respectively^22,25^, and share 70.6% identity and 82.3% similarity at the amino acid level. Subsequently, PIS, a 213-amino acid membrane enzyme, catalyses the synthesis of PI from *myo*-inositol and CDP-DAG^21,22^. To examine the effects of PIS, CDS1 and CDS2 overexpression on the metabolism of cellular phospholipids, we established HEK293 cell lines stably expressing FLAG-PIS (HEK/FLAG-PIS), FLAG-CDS1 (HEK/FLAG-CDS1) or FLAG-CDS2 (HEK/FLAG-CDS2). A FLAG-tag was fused to the N-terminus of each enzyme for immunodetection using an anti-FLAG antibody. On SDS-PAGE, FLAG-tagged PIS, CDS1 and CDS2 were observed as protein bands migrating at ~22 kDa, ~46 kDa and ~45 kDa, respectively, (Figs 3a and S6). Using anti-PIS, anti-CDS1 and anti-CDS2 antibodies, we also detected the expression of these proteins in the stable cell lines. However, in mock-transfected HEK293 (HEK293 mock) cells, endogenous expression of PIS, CDS1 and CDS2 was not detected (Figs 3a and S6). These results raise the possibility that there are other unknown subtypes of PIS and CDS or alternative pathways for PI production in HEK293 cells. PIS, CDS1 and CDS2 are ER-integral membrane proteins. Kim *et al*. previously reported that PIS associates with rapidly moving ER-derived membrane compartments and that CDS1 and CDS2 reside in the tubular ER but not in the PIS-containing compartments^23^. To clarify whether the FLAG-PIS, FLAG-CDS1 and FLAG-CDS2 proteins were localized to the ER, purified microsomal and mitochondrial fractions were isolated from the cell lines. Calnexin (CNX) and cytochrome *c* oxidase subunit IV (COX IV) are well-known markers of the ER and mitochondria, respectively. Immunoblotting with anti-FLAG, anti-CNX and anti-COX IV antibodies showed that FLAG-PIS, FLAG-CDS1 and FLAG-CDS2 were recovered predominantly in the purified microsomal fraction (Figs 3b and S6). In addition, the localization of FLAG-PIS, FLAG-CDS1 and FLAG-CDS2 was analysed by using confocal fluorescence microscopy. The immunofluorescence signals of FLAG-PIS, FLAG-CDS1, and FLAG-CDS2 colocalized with those of CNX, but no localization was found within the mitochondria or nuclei (Supplementary Fig. S7). Taken together, these results suggest that FLAG-PIS, FLAG-CDS1 and FLAG-CDS2 are mainly localized in the ER. Furthermore, we evaluated whether overexpression of PIS, CDS1 or CDS2 affected the growth of HEK293 cells. The exponential increase in cell density is shown in Fig. 3c. The doubling times between days 1 and 6 were not significantly different among HEK293 mock, HEK/FLAG-PIS, HEK/FLAG-CDS1 and HEK/FLAG-CDS2 cells (35.03 ± 1.23, 33.21 ± 0.05, 36.49 ± 0.46, and 35.16 ± 0.38 h, respectively; mean ± S.E., n = 3, one-way ANOVA, *df* = 11, *F* = 3.92, *P* = 0.054).

**Figure 4:**
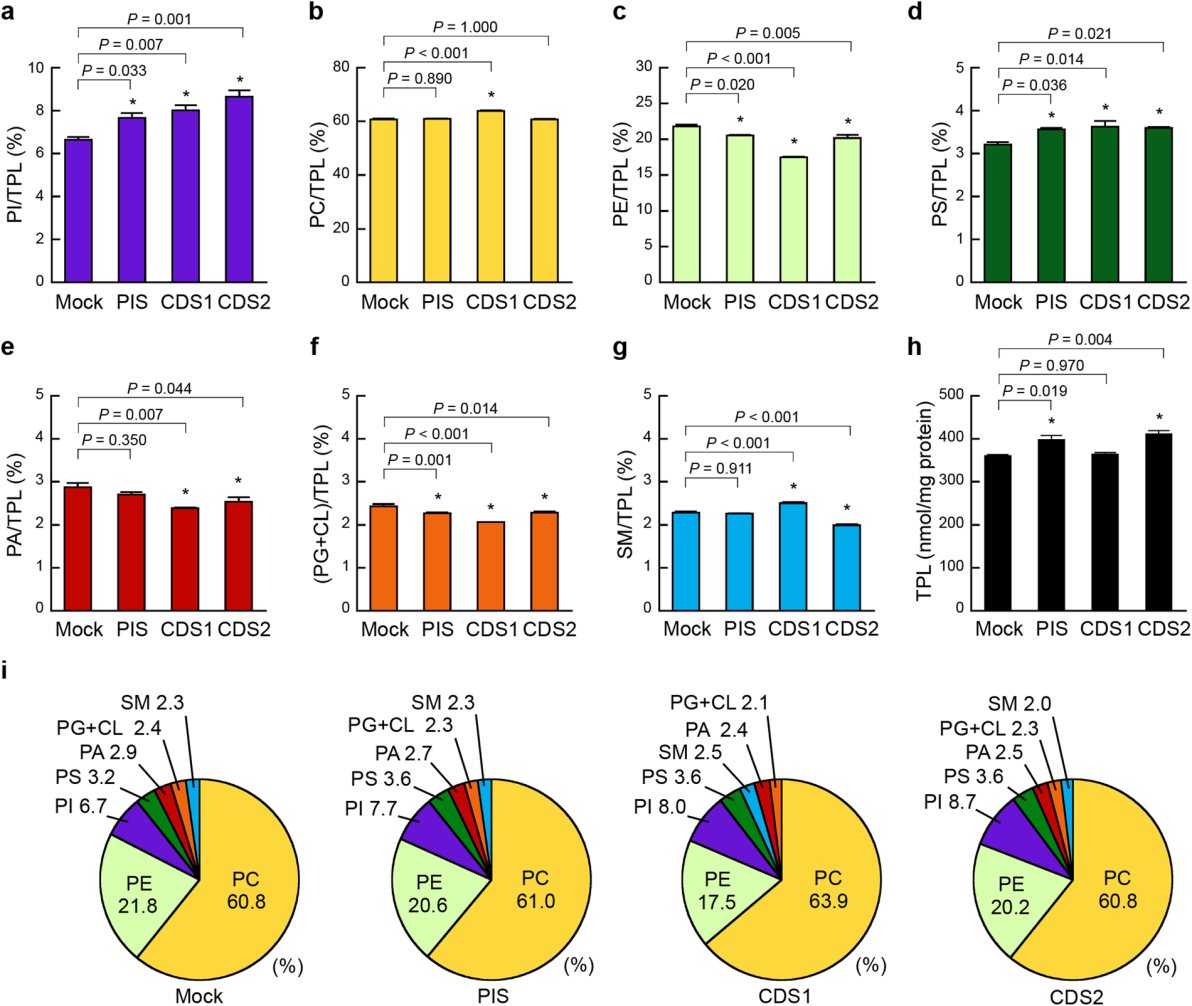
Effects of PIS, CDS1 or CDS2 overexpression on cellular phospholipid composition. HEK293 mock (Mock), HEK/FLAG-PIS (PIS), HEK/FLAG-CDS1 (CDS1) and HEK/FLAG-CDS2 (CDS2) cells were seeded in 10-cm dishes at a density of 5.0 × 10^6^ cells, and cultured in MEM containing 10% FBS for 48 h at 37 °C. Then, the cells were incubated with MEM supplemented with 0.02% BSA for 18 h at 37 °C. After incubation, cellular phospholipids were extracted. There was no significant difference in the cell densities among these cell lines (HEK293 mock, 23.94 ± 1.67; HEK/FLAG-PIS, 23.08 ± 1.39; HEK/FLAG-CDS1, 25.61 ± 1.11; HEK/FLAG-CDS2, 23.69 ± 1.80 μg protein/cm^2^). The total phospholipid (TPL) content (**h**) was calculated as the sum of PI, PC, PE, PS, PA, PG + CL and SM contents. The ratios of PI/TPL (**a**), PC/TPL (**b**), PE/TPL (**c**), PS/TPL (**d**), PA/TPL (**e**), (PG + CL)/TPL (**f**) and SM/TPL (**g**) in the cells were determined by the enzymatic measurements (mean ± S.E., n = 3, *P < 0.05, significantly different from HEK293 mock cells, one-way ANOVA followed by Dunnett’s test, *df* = 11, (**a**) *F* = 13.46, (**b**) *F* = 30.47, (**c**) *F* = 53.17, (**d**) *F* = 6.11, (**e**) *F* = 6.85, (**f**) *F* = 26.48, (**g**) *F* = 88.13, (**h**) *F* = 11.17). (**i**) The phospholipid compositions in the indicated cell lines are shown as pie charts.

It has been reported that transient overexpression of PIS or CDS1 in COS-7 cells results in an 8.2% or 15.8% increase in PI labelling, respectively, by metabolically labelling cells with [^3^H]-inositol and measuring the radioactivity of PI following TLC separation^22^. It has also been demonstrated, by labelling HEK293 cells with [^3^H]-inositol, that downregulation of endogenous PIS by RNAi reduces the level of radiolabelled PI^23^. These findings suggest that the cellular content of PI depends on the expression levels of these enzymes. To ascertain whether the alteration in the cellular contents of phospholipid classes can be detected using our enzymatic methods, we analysed the phospholipid compositions in HEK293 mock, HEK/FLAG-PIS, HEK/FLAG-CDS1 and HEK/FLAG-CDS2 cells at similar cell densities (23.94 ± 1.67, 23.08 ± 1.39, 25.61 ± 1.11, and 23.69 ± 1.80 μg protein/cm^2^, respectively; mean ± S.E., n = 3, one-way ANOVA, *df* = 11, *F* = 0.51, *P* = 0.685). The TPL content in HEK/FLAG-PIS or HEK/FLAG-CDS2 cells was slightly but significantly higher than that in HEK293 mock cells, whereas that in HEK/FLAG-CDS1 cells was unchanged (Fig. 4h). Compared with HEK293 mock cells, HEK/FLAG-PIS, HEK/FLAG-CDS1 and HEK/FLAG-CDS2 cells exhibited 1.15-, 1.21- and 1.30-fold increases in the PI/TPL ratio, respectively (Fig. 4a), likely due to the enhancement of PI production by PIS, CDS1 or CDS2 overexpression. The PC/TPL ratio in HEK/FLAG-CDS1 cells was unexpectedly high compared with that in HEK293 mock cells (Fig. 4b). Interestingly, FLAG-PIS, FLAG-CDS1 or FLAG-CDS2 expression decreased the PE/TPL ratio but increased the PS/TPL ratio (Fig. 4c,d). The ratio of PA, a substrate of CDS1 or CDS2, to TPL was significantly decreased in HEK/FLAG-CDS1 and HEK/FLAG-CDS2 cells but not in HEK/FLAG-PIS cells (Fig. 4e). FLAG-PIS, FLAG-CDS1 or FLAG-CDS2 expression slightly reduced the (PG + CL)/TPL ratio (Fig. 4f). The SM/TPL ratio was higher in HEK/FLAG-CDS1 cells but lower in HEK/FLAG-CDS2 cells compared with that in HEK293 mock cells (Fig. 4g). As shown in Fig. 4h, the contents of the phospholipid classes were in the order PC > PE > PI > PS ≥ PA ≥ PG + CL ≥ SM in these cells. It is conceivable that the ratio of each phospholipid class to TPL in cells is regulated to maintain the functions of membrane proteins and the structure of membranes.

### Effects of PIS, CDS1 or CDS2 overexpression on the phospholipid composition in intracellular organelles

**Figure 5:**
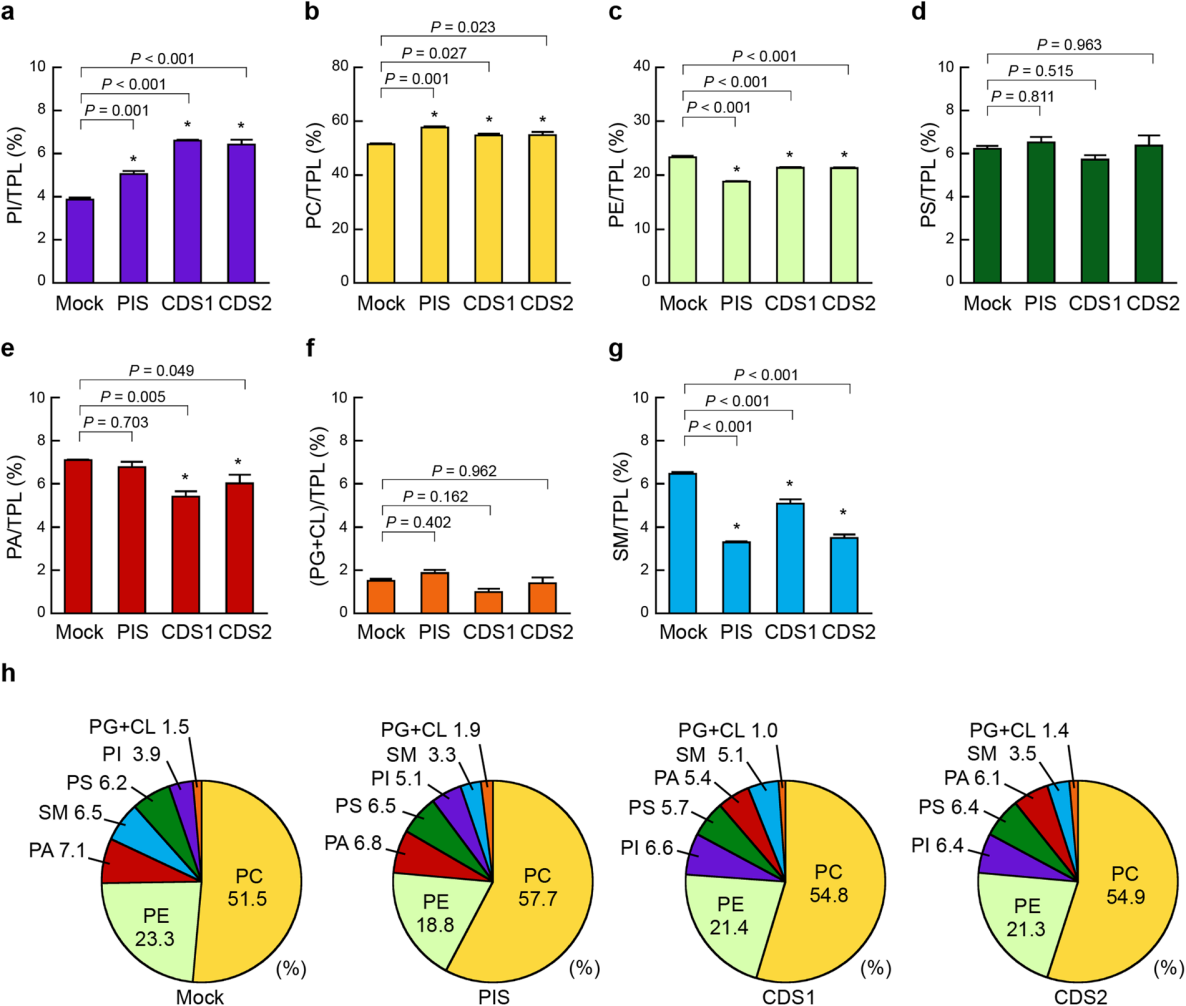
Effects of PIS, CDS1 or CDS2 overexpression on microsomal phospholipid composition. HEK293 mock (Mock), HEK/FLAG-PIS (PIS), HEK/FLAG-CDS1 (CDS1) and HEK/FLAG-CDS2 (CDS2) cells were seeded in 10-cm dishes at a density of 2.0 × 10^7^ cells and were cultured in MEM containing 10% FBS for 48 h at 37 °C. Then, the cells were incubated with MEM supplemented with 0.02% BSA for 18 h at 37 °C. After incubation, purified microsomal fractions were isolated from the cells and microsomal phospholipids were extracted. The total phospholipid (TPL) content was calculated as the sum of PI, PC, PE, PS, PA, PG + CL and SM contents. The ratios of PI/TPL (**a**), PC/TPL (**b**), PE/TPL (**c**), PS/TPL (**d**), PA/TPL (**e**), (PG + CL)/TPL (**f**) and SM/TPL (**g**) in the purified microsomal fractions were determined by the enzymatic measurements (mean ± S.E., n = 3, *P < 0.05, significantly different from HEK293 mock cells, one-way ANOVA followed by Dunnett’s test, *df* = 11, (**a**) *F* = 80.66, (**b**) *F* = 12.59, (**c**) *F* = 106.27, (**d**) *F* = 1.43, (**e**) *F* = 8.41, (**f**) *F* = 4.14, (**g**) *F* = 116.18). (**h**) The phospholipid compositions of the purified microsomal fractions from the indicated cell lines are shown as pie charts.

**Figure 6:**
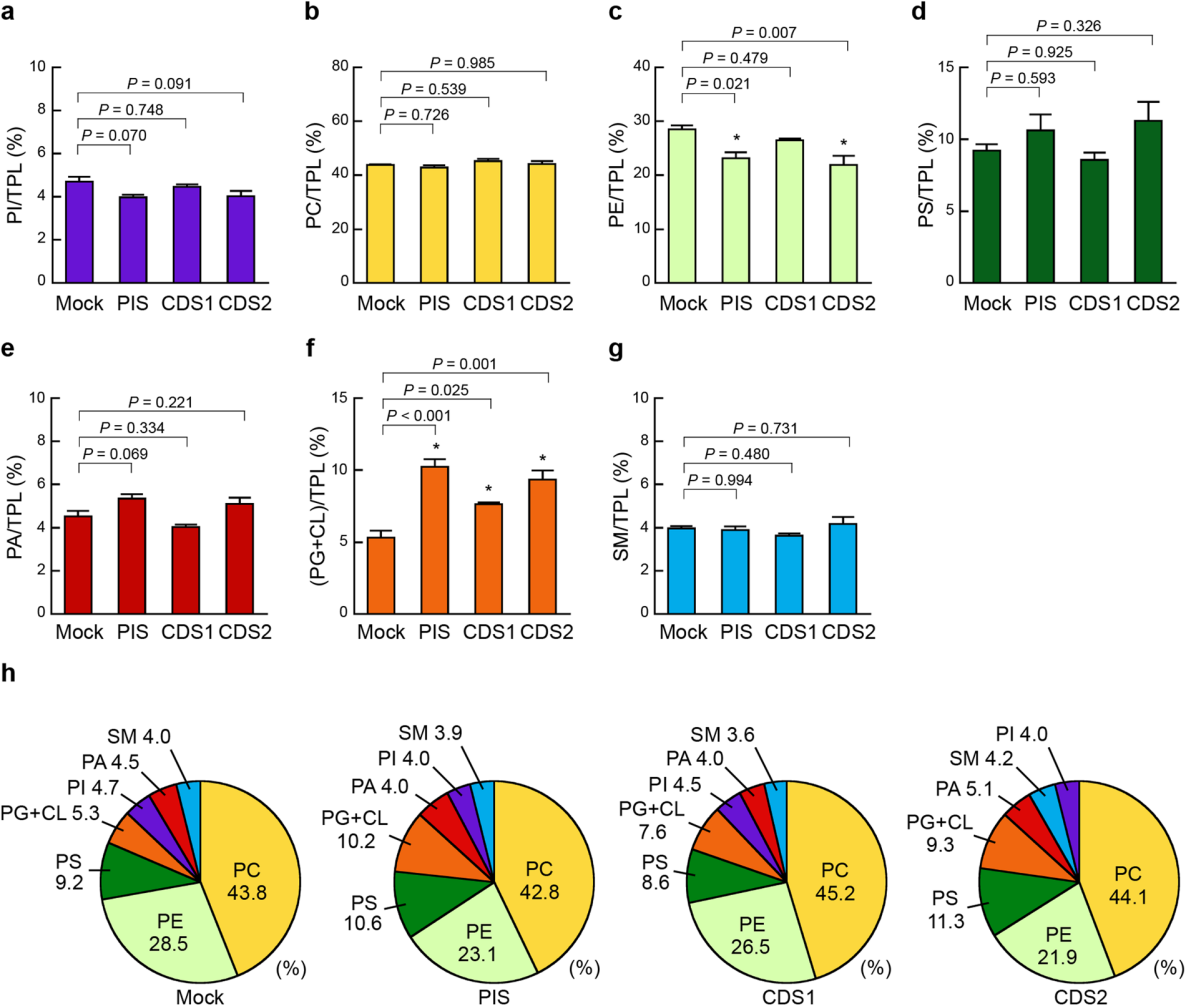
Effects of PIS, CDS1 or CDS2 overexpression on mitochondrial phospholipid composition. HEK293 mock (Mock), HEK/FLAG-PIS (PIS), HEK/FLAG-CDS1 (CDS1) and HEK/FLAG-CDS2 (CDS2) cells were seeded in 10-cm dishes at a density of 2.0 × 10^7^ cells, and cultured in MEM containing 10% FBS for 48 h at 37 °C. Then, the cells were incubated with MEM supplemented with 0.02% BSA for 18 h at 37 °C. After incubation, purified mitochondrial fractions were isolated from the cells and mitochondrial phospholipids were extracted. The total phospholipid (TPL) content was calculated as the sum of PI, PC, PE, PS, PA, PG + CL and SM contents. The ratios of PI/TPL (**a**), PC/TPL (**b**), PE/TPL (**c**), PS/TPL (**d**), PA/TPL (**e**), (PG + CL)/TPL (**f**) and SM/TPL (**g**) in the purified mitochondrial fractions were determined by the enzymatic measurements (mean ± S.E., n = 3, *P < 0.05, significantly different from HEK293 mock cells, one-way ANOVA followed by Dunnett’s test, *df* = 11, (**a**) *F* = 3.32, (**b**) *F* = 1.41, (**c**) *F* = 7.73, (**d)** *F* = 1.83, (**e**) *F* = 7.22, (**f**) *F* = 20.06, (**g**) *F* = 1.54). (**h**) The phospholipid compositions of the purified mitochondrial fractions from the indicated cell lines are shown as pie charts.

The ER and mitochondria are essential sites for the biosynthesis of phospholipids^3,4,7,8^. To understand the control of cellular phospholipid metabolism in detail, we next investigated whether the overexpression of PIS, CDS1 or CDS2 affected the phospholipid composition of the intracellular organelles. As shown in Figs 5 and 6, we characterized the phospholipid compositions of the microsomal and mitochondrial fractions purified from HEK293 mock, HEK/FLAG-PIS, HEK/FLAG-CDS1 and HEK/FLAG-CDS2 cells using our enzymatic fluorometric assays. The PI/TPL ratio in the microsomal membranes was 1.30-, 1.70- and 1.66-fold higher in HEK/FLAG-PIS, HEK/FLAG-CDS1 and HEK/FLAG-CDS2 cells, respectively, than in HEK293 mock cells (Fig. 5a). The microsomal PA/TPL ratio was significantly decreased in HEK/FLAG-CDS1 and HEK/FLAG-CDS2 cells but not in HEK/FLAG-PIS cells (Fig. 5e). Additionally, in microsomal membranes, expression of FLAG-PIS, FLAG-CDS1 or FLAG-CDS2 resulted in an increased PC/TPL ratio but in decreased PE/TPL and SM/TPL ratios (Fig. 5b,c,g). However, the PS/TPL and (PG + CL)/TPL ratios in microsomal fractions were not changed by FLAG-PIS, FLAG-CDS1 or FLAG-CDS2 expression (Fig. 5d,f). In the microsomal fraction from HEK293 mock cells, the most abundant phospholipid was PC, followed in descending order by PE, PA, SM, PS, PI and PG + CL (Fig. 5h). However, the proportion of PI was higher than that of SM in HEK/FLAG-PIS cells and higher than those of PA and SM in HEK/CDS1 and HEK/CDS2 cells (Fig. 5h).

In contrast to the findings in the microsomal fractions, expression of FLAG-PIS, FLAG-CDS1 or FLAG-CDS2 did not affect the ratio of PI, PC, PS, PA or SM to TPL in the purified mitochondrial fractions (Fig. 6a,b,d,e,g). Significant increases in the (PG + CL)/TPL ratios were observed in the mitochondrial fractions of HEK/FLAG-PIS, HEK/FLAG-CDS1 and HEK/FLAG-CDS2 cells (Fig. 6f). HEK/FLAG-PIS and HEK/FLAG-CDS2 cells exhibited lower PE/TPL ratios in the mitochondrial fractions than HEK293 mock cells (Fig. 6c), similar to the findings in the microsomal fractions. In the mitochondrial fractions from these cells, the phospholipid proportions, from highest to lowest, were PC > PE > PS > PG + CL > PI ≥ PA ≥ SM (Fig. 6h).

As shown in Figs 5a and 6a, in HEK293 mock cells, the PI/TPL ratios in microsomes and mitochondria were 3.88 ± 0.08% and 4.70 ± 0.24%, respectively. Previous investigations demonstrated that the PI/TPL ratios in microsomes and mitochondria from mouse liver tissues are ~4% and ~7%, respectively^38^, and that, in rat liver tissues, the PI/TPL ratios are 3–10% in microsomes and 1–5% in mitochondria^7,38–41^. Thus, despite the differences in animal species, cell types and experimental conditions, the microsomal and mitochondrial PI/TPL ratios determined by our enzymatic assay were comparable with those from previous reports.

We demonstrated that overexpression of CDS1 or CDS2 led to an increased proportion of PI and a decreased proportion of PA in microsomal membranes (Fig. 5a,e). As PI is the precursor of all PIPs, the generation of PIPs is likely dependent on the intracellular PI level in addition to the activity of PI kinases. PA binds to and activates the mammalian target of rapamycin, which is important for regulating cellular growth^42^. However, the reductions in the proportions of PA in microsomal membranes did not affect the proliferation rate of HEK/FLAG-CDS1 or HEK/FLAG-CDS2 cells (Figs 3c and 5e). In contrast, PIS overexpression induced an increase in the proportion of PI but no change in that of PA in microsomal membranes (Fig. 5e), which may be attributed to the compensation for the loss of PA due to PIS by DAG kinase, PLD or de novo glycerol-3-phosphate acylation.

HEK293 cells do not express PE *N*-methyltransferase and generate PC through the CDP-choline pathway^3,7,13^, where the rate-limiting step is the conversion of phosphocholine to CDP-choline by CTP:phosphocholine cytidylyltransferase (CCT)^3,7^. The activity of CCT is modulated by reversible binding to the ER or nuclear membrane. PI, PS, PA, PG and CL enhance membrane association and activation of CCT^43^. In HEK/FLAG-PIS, HEK/FLAG-CDS1 and HEK/FLAG-CDS2 cells, the increased proportions of PC in microsomal membranes (Fig. 5b) may be partially due to the activation of CCT by PI. SM synthesis involves phosphocholine transfer from PC to ceramide by SM synthase^44^. In microsomal membranes, overexpression of PIS, CDS1 or CDS2 resulted in a decrease in the SM proportion despite a concomitant increase in the PC proportion (Fig. 5b,g), which may be due to the decrease in SM synthase activity and/or to the loss of ceramide.

The capacity of mitochondria to generate phospholipids is restricted to PG, CL, PE and PA; thus, mitochondrial import of PI, PC, PS and SM is very important for various mitochondrial processes^7,8^. In this study, overexpression of PIS, CDS1 or CDS2 caused no change in the PI, PC, PS and SM proportions in mitochondrial membranes (Fig. 6a,b,d,g), suggesting that mitochondrial import of these phospholipid classes is strictly regulated, even if their levels are altered in the ER. Recent studies found that TAMM41 is implicated in mitochondrial CDS activity^45^. In mammalian mitochondria, phosphatidylglycerophosphate synthase 1 catalyses the conversion of CDP-DAG to phosphatidylglycerophosphate, which is rapidly dephosphorylated to produce PG^7,8^. By the action of CL synthase, CL is generated from CDP-DAG and PG at the mitochondrial inner membrane. We showed that overexpression of CDS1 or CDS2 elevated the PG + CL proportion in mitochondrial membranes but did not affect the PA proportion (Fig. 6e,f), suggesting that CDP-DAG molecules transferred from the ER into mitochondria were used for PG and CL synthesis.

In culture medium lacking ethanolamine, PE is generated mainly through the PS decarboxylation pathway in mitochondria^4^. Overexpression of PIS or CDS2 reduced the PE proportions in both mitochondrial and microsomal membranes (Figs 5c and 6c), which may be due to decreased PS decarboxylase activity in mitochondria. Altogether, these results indicate that overexpression of PIS, CDS1 or CDS2 affects not only the production of PI but also the metabolism of other phospholipid classes in the ER and mitochondria.

Recently, several PI transfer proteins have been reported^46^. N-terminal domain-interacting receptor 2 (Nir2) is a PI transfer protein implicated in the exchange of PI and PA at ER-plasma membrane contact sites^47,48^. Oxysterol-binding protein-related protein 5 (ORP5) and ORP8 act as PS/PI(4)P counterexchangers at the contact sites between the ER and plasma membranes^49,50^. Thus, Nir2, ORP5 or ORP8 may function in the distribution of PI, PA or PS between the ER and plasma membranes and in the maintenance of phospholipid homeostasis in PIS, CDS1 or CDS2-overexpressing cells.

In conclusion, we developed and validated a new enzyme-based fluorometric method for quantifying PI to complement our previously developed assays for PC, PE, PS, PA, PG + CL and SM. These enzymatic assays make it possible to quantify all major phospholipid classes in cultured cells and intracellular organelles. All enzymes and substrates used in these assays are commercially available. These enzymatic fluorometric methods have high accuracy and high sensitivity and can process many samples in parallel. The usefulness of these procedures was confirmed using cells and purified intracellular organelles. By performing comprehensive analyses of major phospholipid classes, we demonstrated that the cellular contents of phospholipid classes were largely dependent on cell density and that overexpression of PIS, CDS1 or CDS2 affected the phospholipid composition of microsomal and mitochondrial membranes. Furthermore, these simple methods may be applicable to animal tissues, fluids, lipoproteins and extracellular vesicles. Thus, our enzymatic fluorometric methods for quantifying phospholipid classes will be helpful in understanding the biological functions of phospholipids in various organisms.

## Methods

### Materials

PLD from *S. chromofuscus* was obtained from Asahi Kasei Pharma (Tokyo, Japan). IDH from *Bacillus subtilis* was purchased from Megazyme (Bray, Ireland). NADH oxidase from *Bacillus licheniformis* was purchased from Sanyo Fine (Osaka, Japan). Peroxidase from horseradish roots was purchased from Oriental Yeast (Tokyo, Japan). NAD^+^ and G418 disulfate were obtained from Nacalai Tesque (Kyoto, Japan). Amplex Red Reagent and Amplex Red Stop Regent were obtained from Molecular Probes (Eugene, OR, USA). Triton X-100 was purchased from Roche Diagnostics (Mannheim, Germany). PI sodium salt from bovine liver, PI sodium salt from soy, DOPI sodium salt, POPI sodium salt, LPI sodium salt from bovine liver, DOPI(3)P diammonium salt, PI(4)P diammonium salt from porcine brain, DOPI(5)P diammonium salt, DOPI(3,4)P_2_ triammonium salt, DOPI(3,5)P_2_ triammonium salt, PI(4,5)P_2_ triammonium salt from porcine brain, DOPI(3,4,5)P_3_ tetraammonium salt and all other phospholipids were obtained from Avanti Polar Lipids (Alabaster, AL, USA). All other chemicals used were of the highest reagent grade.

### Enzymatic measurement of PI

In Fig. 1a, the enzymatic steps for PI quantification are depicted. Reagent I1 contained 200 U/ml PLD, 2.4 mM CaCl_2_, 50 mM NaCl and 50 mM Tris-HCl (pH 7.4). Reagent I2 contained 25 U/ml IDH, 10 mM NAD^+^, 150 mM NaCl and 150 mM Tris-HCl (pH 7.4). Regent I3 contained 1 U/ml NADH oxidase, 6.25 U/ml peroxidase, 187.5 μM Amplex Red, 0.125% Triton X-100, 50 mM NaCl and 50 mM Tris-HCl (pH 7.4). Standard solutions of PI were dissolved in 1% Triton X-100 aqueous solution.

Reagent I1 (10 μl) was added to the samples (10 μl) and incubated at 37 °C for 1 h. Then, PLD was heat-inactivated by 3-min incubation at 96 °C, and the denatured enzyme was removed by centrifugation for 5 min at 7,200 *g*. The supernatant (10 μl) was mixed with Reagent I2 (10 μl) and incubated at 25 °C for 2 h. Then, 80 μl of Reagent I3 was added. After a 1-h incubation at 45 °C, 20 μl of Amplex Red Stop Reagent was added. Fluorescence intensity was measured at 544 nm (excitation) and 590 nm (emission) by an Infinite M200 multimode microplate reader (Tecan, Männedorf, Switzerland).

### Plasmid construction

The human PIS gene (GenBank: NM_006319), the human CDS1 gene (GenBank: NM_001263) and the human CDS2 gene (GenBank: NM_003818) were obtained from Kazusa DNA Research Institute (Kisarazu, Japan). An oligonucleotide encoding the FLAG (DYKDDDDK) epitope was appended to the 5′ end of the genes via PCR. Plasmids for FLAG-PIS, FLAG-CDS1 or FLAG-CDS2 expression were constructed by inserting each PCR product into the pIRESneo3 mammalian expression vector (Clontech, Mountain View, CA, USA), which promotes the establishment of pools of stably transfected cells^51^.

### Cell culture and establishment of stable transformants

HEK293 cells were cultured in 5% CO_2_ at 37 °C in DMEM containing 10% heat-inactivated foetal bovine serum (FBS)^19^. Lipofectamine Reagent and PLUS Reagent (Invitrogen, Carlsbad, CA, USA) were used to transfect cells with pIRESneo3 (mock), pIRESneo3/FLAG-PIS, pIRESneo3/FLAG-CDS1 or pIRESneo3/FLAG-CDS2. Cells were selected using 1.2 mg/ml G418, and a large number of drug-resistant clones were pooled in one dish. Expression of FLAG-PIS, FLAG-CDS1 and FLAG-CDS2 was assessed by immunoblotting.

### Immunoblotting

Cells were sonicated and lysed with 1% Triton X-100 in PBS to prepare whole cell lysates. Mitochondrial and microsomal fractions were isolated as previously reported^52^ and were lysed with 1% Triton X-100 in 5 mM HEPES buffer (pH 7.4). Samples were separated on 7%, 10% or 15% polyacrylamide gels by SDS-PAGE calibrated with Precision Plus Protein WesternC Standards (Bio-Rad Laboratories, Hercules, CA, USA) and were transferred to PVDF membranes (Merck Millipore, Billerica, MA). Membranes were blocked with Blocking One (Nacalai Tesque), and were then immunoblotted with monoclonal anti-FLAG antibody M2 (1:2,000 dilution; Sigma-Aldrich, St. Louis, MO, USA), polyclonal anti-PIS antibody (1:2,000; Atlas Antibodies, Bromma, Sweden), monoclonal anti-CDS1 antibody 2D10 (1:3,000; Sigma-Aldrich), polyclonal anti-CDS2 antibody (1:6,000; Proteintech, Rosemont, IL, USA), monoclonal anti-β-actin antibody AC-15 (1:1,000; Sigma-Aldrich), monoclonal anti-COX IV antibody 3E11 (1:10,000; Cell Signaling Technology, Danvers, MA, USA) or polyclonal anti-CNX antibody (1:2,000; Stressgen, Ann Arbor, MI, USA), followed by incubation with horseradish peroxidase (HRP)-conjugated goat anti-mouse IgG (1:3,000; Invitrogen), HRP-conjugated goat anti-rabbit IgG (1:3,000; Merck Millipore) or Precision Protein StrepTactin-HRP conjugate (1:10,000; Bio-Rad). The antibodies were diluted in Can Get Signal Immunoreaction Enhancer Solution (Toyobo, Osaka, Japan) just before use. Protein-antibody complexes were detected using Amersham ECL Prime Western Blotting Detection Reagent and an ImageQuant LAS 4000 mini biomolecular imager (GE Healthcare, Buckinghamshire, UK).

### Measurement of phospholipid contents in HEK293 cells

To measure the contents of cellular phospholipids, cells were subcultured and grown in MEM supplemented with 10% FBS in 10-cm dishes at varying cell densities. After a 48-h incubation, cells were washed and incubated in MEM containing 0.02% bovine serum albumin (BSA) for 18 h at 37 °C. Cells were washed, scraped with cold PBS and sonicated using an Ultrasonic Disruptor UR-20P (Tomy Seiko, Tokyo, Japan) to prepare cell homogenates. To measure the contents of phospholipids in purified mitochondrial and microsomal fractions, cells were subcultured at a density of 2.0 × 10^7^ cells in MEM supplemented with 10% FBS in 10-cm dishes. After a 48-h incubation, cells were washed and incubated with MEM containing 0.02% BSA for 18 h at 37 °C. Then, purified mitochondrial and microsomal fractions were isolated according to the previously described method^52^. The fractions were suspended in 5 mM HEPES buffer (pH 7.4). The concentrations of protein in cell homogenates and in purified mitochondrial and microsomal fractions were determined using a BCA protein assay kit (Thermo Scientific, Rockford, IL, USA). Phospholipids in cells or organelles were extracted by the method of Folch^31,36,53^ and dissolved in 1% Triton X-100. The PI, PC, PE, PS, PA, PG + CL and SM contents in the extracts were quantified by enzymatic methods^13,16–19^. The total phospholipid (TPL) content was calculated as the sum of PI, PC, PE, PS, PA, PG + CL and SM contents.

### Phospholipid extraction

Phospholipids were extracted from the samples by the method of Folch^31,36,53^. In brief, the sample solution (1.0 ml) was added to 4.0 ml of chloroform/methanol (2:1) solution and vortexed. After vortexing, the sample was incubated overnight at 4 °C. Phase separation was completed by centrifugation. The upper aqueous phase and the interfacial material were carefully removed. The recovered lower organic phase was washed with 1 ml of H_2_O. The aqueous phase was removed again, and the organic solvent was evaporated from the lower phase. The evaporated sample was dissolved by the addition of 1% Triton X-100 (200 μl).

### Statistical analysis

Multiple comparisons were performed using Dunnett’s test following one-way ANOVA. Differences were considered significant at *P* < 0.05 (two-tailed). The degrees of freedom (*df*) and F-values (*F*) are noted in the figure legends. The results are given as the means ± S.E.s (n = 3 biologically independent experiments) unless otherwise indicated.

## Supplementary information

Supplementary Information
