# Supplementary material for: Enzymatic fluorometric assays for quantifying all major phospholipid classes in cells and intracellular organelles
Source: Sci Rep. 2019 Jun 13;9:8607. doi: 10.1038/s41598-019-45185-0 (PMC6565719; doi:10.1038/s41598-019-45185-0)
Supplement: Supplementary file 1 — Supplementary Information [file 41598_2019_45185_MOESM1_ESM.pdf]

Supplementary Information

Enzymatic fluorometric assays for quantifying all major phospholipid classes in cells and intracellular organelles

Tokuji Tsuji, Shin-ya Morita*, Yoshito Ikeda, Tomohiro Terada

Department of Pharmacy, Shiga University of Medical Science Hospital, Otsu City, Shiga 520-2192, Japan

*Correspondence and requests for materials should be addressed to S.-y.M. (email: smorita@belle.shiga-med.ac.jp)

Supplementary Methods

Materials.

1,2-dioleoyl PI(4)P ammonium salt was purchased from Avanti Polar Lipids. *myo*-Inositol and NADH disodium salt were obtained from Nacalai Tesque. H_2O_2 was purchased from FUJIFILM Wako Pure Chemical. All other chemicals used were of the highest reagent grade.

myo-Inositol measurement.

Enzymatic assays have been widely employed to measure the concentration of *myo*-inositol [1, 2]. We modified the method for the enzymatic measurement of *myo*-inositol using Amplex Red. A three-reagent system was used for measurement. *myo*-Inositol standard solutions were dissolved in water. Reagent I2 (10 μl) was added to the sample (10 μl) and incubated at room temperature for 2 h. Then, Reagent I3 (80 μl) was added. After a 1-h incubation at 45ºC, Amplex Red Stop Reagent (20 μl) was added. The fluorescence intensity (excitation 544 nm, emission 590 nm) was measured using a multimode microplate reader Infinite M200 (Tecan).

NADH measurement.

We slightly modified the enzymatic fluorometric assay for quantifying NADH reported by Batchelor and Zhou [3]. The reaction buffer contained 50 mM NaCl and 50 mM Tris-HCl (pH 7.4). NADH standard solutions were dissolved in water. The reaction buffer (10 μl) and Reagent I3 (80 μl) were added to the sample (10 μl). After a 1-h incubation at 45ºC, Amplex Red Stop Reagent (20 μl) was added. The fluorescence intensity (excitation 544 nm, emission 590 nm) was measured using a microplate reader.

H_2O_2 measurement.

Enzymatic fluorometric assays have been widely employed to measure the concentration of H_2O_2 [4, 5]. Reagent HP contained 5 U/ml peroxidase, 300 μM Amplex Red, 0.2% Triton X-100, 50 mM NaCl and 50 mM Tris-HCl (pH 7.4). H_2O_2 standard solutions were dissolved in water. The reaction buffer (40 μl) and Reagent HP (50 μl) were added to the sample (10 μl). After a 30-min incubation at room temperature,

Amplex Red Stop Reagent (20 μl) was added. The fluorescence intensity (excitation 544 nm, emission 590 nm) was measured using a microplate reader.

PI(4)P measurement.

1,2-dioleoyl PI(4)P was dissolved in water. The solution containing PI(4)P was processed by the extraction method of Folch [6-8], and the extract was then dissolved in 1% Triton X-100. The concentration of PI(4)P in the sample with or without lipid extraction was measured by the enzymatic fluorometric assay of PI.

Differential interference contrast imaging.

HEK293 cells were cultured in 10% FBS-containing MEM in 10-cm dishes at varying cell densities for 48 h. Cells were washed and incubated with MEM containing 0.02% BSA for 18 h. Differential interference contrast images were captured by TMD300 light microscopy with the Plan 20×/0.40 objective lens (Nikon, Tokyo, Japan) and Retiga 2000R CCD camera (Teledyne QImaging, Surrey, BC, Canada) using Image-Pro Plus J software (Media Cybernetics, Rockville, MD, USA).

Immunofluorescence staining and confocal imaging.

Cells were seeded on poly-L-lysine-coated glass cover slips and cultured in 10% FBS-containing MEM for 48 h. For fluorescence staining of mitochondria, cells on cover slips were incubated with phenol red-free MEM containing 0.02% BSA and 250 nM MitoTracker Red CMXRos (Molecular Probes) for 45 min at 37°C before immunostaining. For immunostaining, cells on cover slips were fixed with 4% paraformaldehyde-containing PBS for 15 min, permeabilized with methanol chilled at -20°C for 15 min, and incubated in 4% BSA-containing PBS for 1 h. Then, cells were incubated with fluorescein isothiocyanate (FITC)-conjugated anti-FLAG mouse monoclonal antibody M2 (1:100, Sigma-Aldrich) and rabbit polyclonal anti-CNX antibody (1:200, Stressgen) for 2 h at room temperature in 1% BSA-containing PBS, and subsequently with Alexa Fluor 568-conjugated goat anti-rabbit IgG antibody (1:200, Molecular Probes) for 1 h at room temperature in 1% BSA-containing PBS. Cells were then incubated with 300 nM 4’, 6-diamidino-2-phenylindole (DAPI) (Sigma-Aldrich) for 5 min. Cover slips were mounted on glass slides using SlowFade Diamond Antifade Mountant (Molecular Probes). Fluorescent images were collected

by TCS SP8 X confocal laser scanning microscopy with the PL Apo 63×/1.20 water-immersion objective lens using LAS X software (Leica Microsystems, Wetzlar, Germany).

Statistical analysis.

Statistical significance was determined using one-way ANOVA followed by Dunnett's test. Differences were considered significant at $P < 0.05$ (two-tailed). Degrees of freedom (*df*) and F-values (*F*) are noted in Figure Legends.

References

1. MacGregor, L.C. & Matschinsky, F.M. An enzymatic fluorimetric assay for myo-inositol. *Anal. Biochem.* **141**, 382-389 (1984).
2. Ashizawa, N., Yoshida, M. & Aotsuka, T. An enzymatic assay for myo-inositol in tissue samples. *J. Biochem. Biophys. Methods* **44**, 89-94 (2000).
3. Batchelor, R.H. & Zhou, M. A resorufin-based fluorescent assay for quantifying NADH. *Anal. Biochem.* **305**, 118-119 (2002).
4. Zhou, M., Diwu, Z., Panchuk-Voloshina, N. & Haugland, R.P. A stable nonfluorescent derivative of resorufin for the fluorometric determination of trace hydrogen peroxide: applications in detecting the activity of phagocyte NADPH oxidase and other oxidases. *Anal. Biochem.* **253**, 162-168 (1997).
5. Mohanty, J.G., Jaffe, J.S., Schulman, E.S. & Raible, D.G. A highly sensitive fluorescent micro-assay of H2O2 release from activated human leukocytes using a dihydroxyphenoxazine derivative. *J. Immunol. Methods* **202**, 133-141 (1997).
6. Folch, J., Lees, M. & Sloane Stanley, G.H. A simple method for the isolation and purification of total lipides from animal tissues. *J. Biol. Chem.* **226**, 497-509 (1957).
7. Wakelam, M.J., Pettitt, T.R. & Postle, A.D. Lipidomic analysis of signaling pathways. *Methods Enzymol.* **432**, 233-246 (2007).
8. Abe-Dohmae, S. et al. Characterization of apolipoprotein-mediated HDL generation induced by cAMP in a murine macrophage cell line. *Biochemistry* **39**, 11092-11099 (2000).

Added (µM)	Measured (µM)	Expected (µM)	Recovery (%)
0	61.5		
25	85.4	86.5	98.7
50	112.4	111.5	100.8
100	161.9	161.5	100.3
250	309.1	311.5	99.2

Supplementary Table S1. Recovery of PI added to the cellular lipid extract. The liver PI standard solution was added to the lipid extract from HEK293 cells. The concentration of PI was measured by the enzymatic assay.

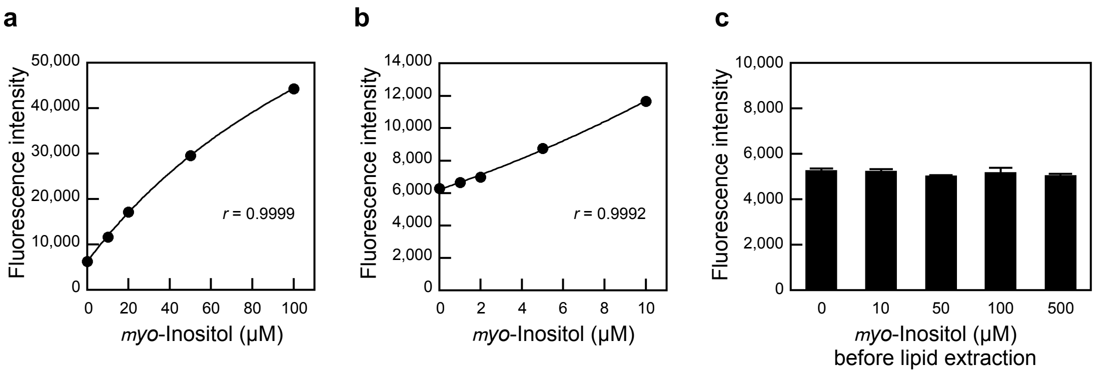

Supplementary Figure S1. Removal of *myo*-inositol by lipid extraction. (**a**) and (**b**) Standard curves for *myo*-inositol measurement. The background fluorescence was 6290. Each point represents the mean of duplicate measurements. The lines were obtained by hyperbolic regression analysis at higher concentrations (r = 0.9999) (**a**) and by quadratic regression analysis at lower concentrations (r = 0.9992) (**b**). (**c**) Fluorescence changes in response to the *myo*-inositol solutions after the lipid extraction by the method of Folch. Before the lipid extraction, the solutions contained the indicated concentrations of *myo*-inositol. There was no significant difference in the fluorescence intensities among these samples (mean ± S.D., n = 3, one-way ANOVA, df = 14, F = 1.95, P = 0.179).

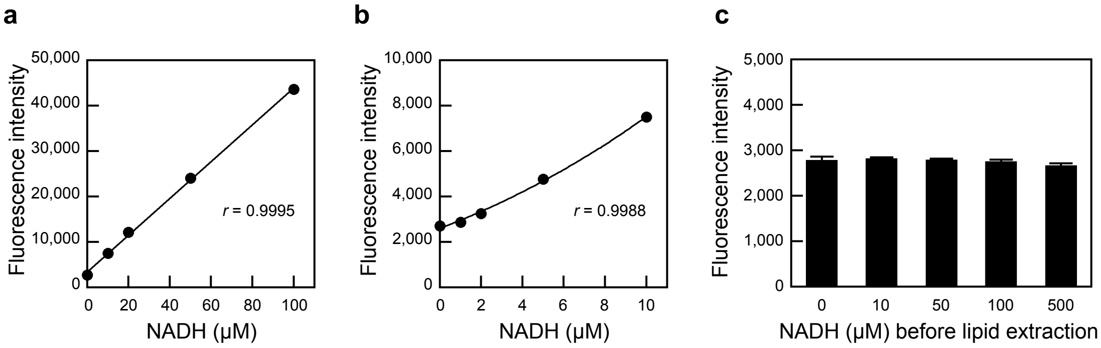

Supplementary Figure S2. Removal of NADH by lipid extraction. (**a**) and (**b**) Standard curves for NADH measurement. The background fluorescence was 2717. Each point represents the mean of duplicate measurements. The lines were obtained by linear regression analysis at higher concentrations (r = 0.9995) (**a**) and by quadratic regression analysis at lower concentrations (r = 0.9988) (**b**). (**c**) Fluorescence changes in response to the NADH solutions after the lipid extraction by the method of Folch. Before the lipid extraction, the solutions contained the indicated concentrations of NADH. There was no significant difference in the fluorescence intensities among these samples (mean ± S.D., n = 3, one-way ANOVA, df = 14, F = 2.47, P = 0.112).

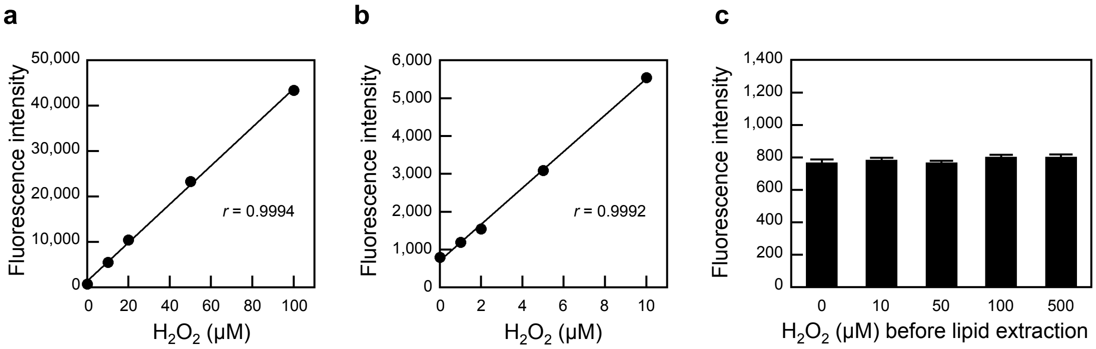

Supplementary Figure S3. Removal of H_2O_2 by lipid extraction. (**a**) and (**b**) Standard curves for H_2O_2 measurement. The background fluorescence was 803. Each point represents the mean of duplicate measurements. The lines were obtained by linear regression analysis. The correlation coefficients were r = 0.9994 at higher concentrations (**a**) and r = 0.9992 at lower concentrations (**b**). (**c**) Fluorescence changes in response to the H_2O_2 solutions after the lipid extraction by the method of Folch. Before the lipid extraction, the solutions contained the indicated concentrations of H_2O_2. There was no significant difference in the fluorescence intensities among these samples (mean ± S.D., n = 3, one-way ANOVA, df = 14, F = 1.96, P = 0.178).

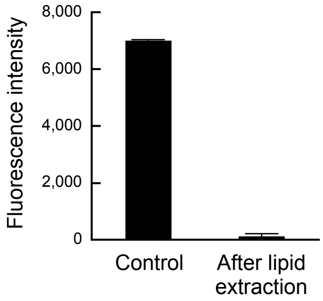

Supplementary Figure S4. Removal of PI(4)P by lipid extraction. In the enzymatic assay of PI, the solution containing 25 μM PI(4)P (control) increased the fluorescence intensity, but the solution after the lipid extraction by the method of Folch did not (mean ± S.D., n =3). The background fluorescence was 10,265.

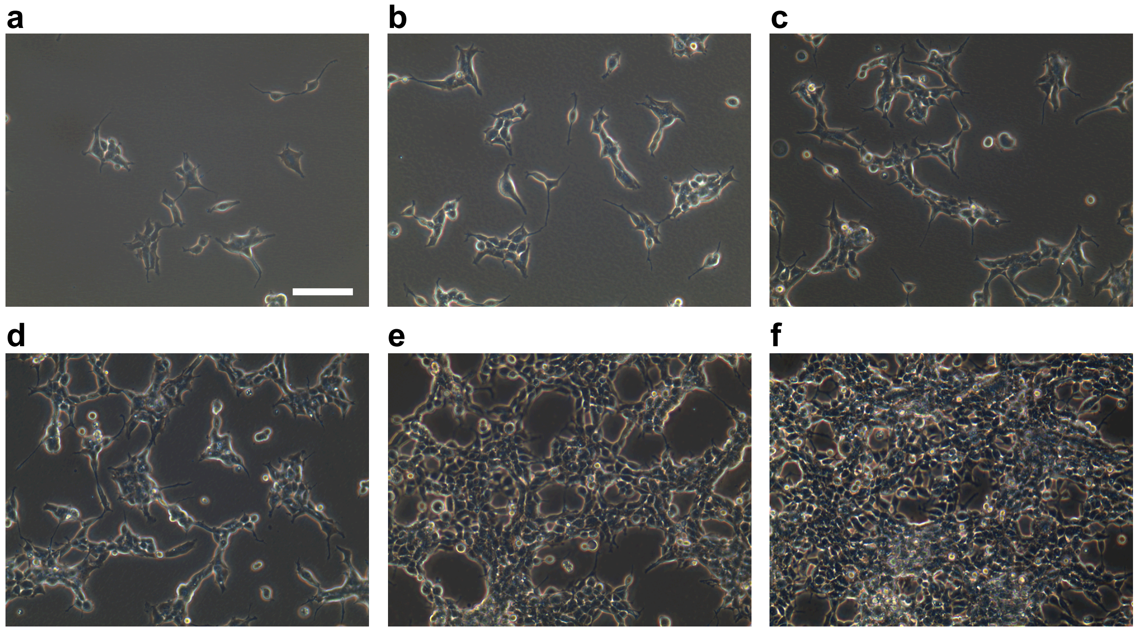

Supplementary Figure S5. Differential interference contrast images of HEK293 cells at varying cell densities. The cell densities were 2.91 (**a**), 5.72 (**b**), 9.24 (**c**), 16.87 (**d**), 46.46 (**e**) and 79.75 μg protein/cm^2 (**f**), respectively. The bar represents 100 μm.

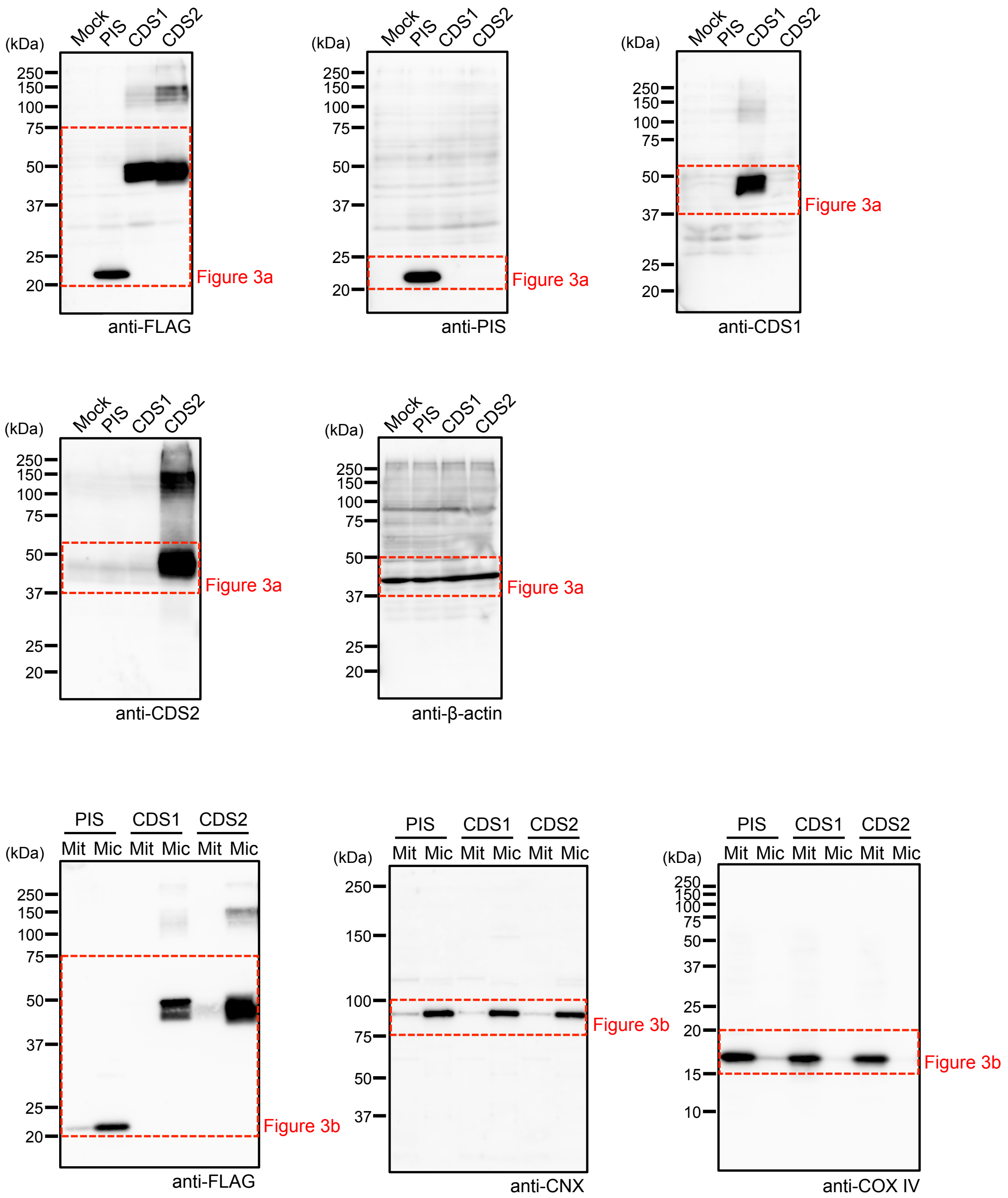

Supplementary Figure S6. Full length blots of Figure 3a and 3b.

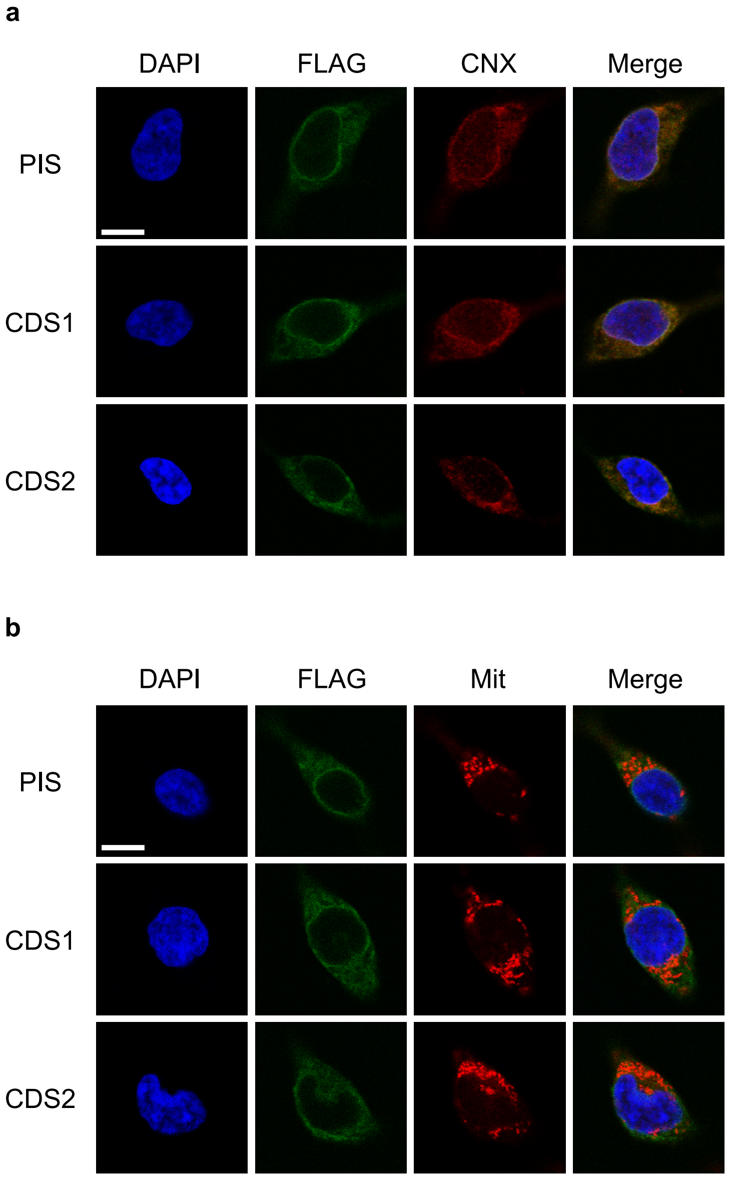

Supplementary Figure S7. Confocal fluorescence imaging of FLAG-PIS, FLAG-CDS1 and FLAG-CDS2. HEK/FLAG-PIS (PIS), HEK/FLAG-CDS1 (CDS1) and HEK/FLAG-CDS2 (CDS2) cells were fixed with 4% paraformaldehyde and permeabilized with methanol. FLAG-tagged PIS, CDS1 or CDS2 was visualized with the FITC-conjugated anti-FLAG mouse monoclonal antibody M2 (green). Nuclei were stained with DAPI (blue). (**a**) The ER marker CNX was immunostained with rabbit polyclonal anti-CNX antibody followed by Alexa568-conjugated secondary antibody (red). (**b**) Mitochondria were stained with MitoTracker Red CMXRos (red). The bar represents 10 μm.
